# Untargeted metabolomics in primary murine bone marrow stromal cells reveals distinct profile throughout osteoblast differentiation

**DOI:** 10.1007/s11306-021-01829-9

**Published:** 2021-09-18

**Authors:** Biswapriya B. Misra, Shobana Jayapalan, Alison K. Richards, Ron C. M. Helderman, Elizabeth Rendina-Ruedy

**Affiliations:** 1 Pine-211, Raintree Park Dwaraka Krishna, Namburu, 522508 Andhra Pradesh India; 2 Maine Medical Center Research Institute, Scarborough, ME USA; 3 Department of Medicine, Division of Clinical Pharmacology, Vanderbilt University Medical Center, Nashville, TN USA; 4 Department of Molecular Physiology and Biophysics, Vanderbilt University, Nashville, TN USA

**Keywords:** Bone, Metabolism, Bioenergetics, ATP, Metabolomics, LC–MS/MS

## Abstract

**Introduction:**

Skeletal homeostasis is an exquisitely regulated process most directly influenced by bone resorbing osteoclasts, bone forming osteoblasts, and the mechano-sensing osteocytes. These cells work together to constantly remodel bone as a mechanism to prevent from skeletal fragility. As such, when an individual experiences a disconnect in these tightly coupled processes, fracture incidence increases, such as during ageing, gonadal hormone deficiency, weightlessness, and diabetes. While therapeutic options have significantly aided in the treatment of low bone mineral density (BMD) or osteoporosis, limited options remain for anabolic or bone forming agents. Therefore, it is of interest to continue to understand how osteoblasts regulate their metabolism to support the energy expensive process of bone formation.

**Objective:**

The current project sought to rigorously characterize the distinct metabolic processes and intracellular metabolite profiles in stromal cells throughout osteoblast differentiation using untargeted metabolomics.

**Methods:**

Primary, murine bone marrow stromal cells (BMSCs) were characterized throughout osteoblast differentiation using standard staining protocols, Seahorse XFe metabolic flux analyses, and untargeted metabolomics.

**Results:**

We demonstrate here that the metabolic footprint of stromal cells undergoing osteoblast differentiation are distinct, and while oxidative phosphorylation drives adenosine triphosphate (ATP) generation early in the differentiation process, mature osteoblasts depend on glycolysis. Importantly, the intracellular metabolite profile supports these findings while also suggesting additional pathways critical for proper osteoblast function.

**Conclusion:**

These data are the first of their kind to characterize these metabolites in conjunction with the bioenergetic profile in primary, murine stromal cells throughout osteoblast differentiation and provide provocative targets for future investigation.

**Supplementary Information:**

The online version contains supplementary material available at 10.1007/s11306-021-01829-9.

## Introduction

Bone is an incredibly dynamic tissue that undergoes continuous remodeling involving bone resorbing osteoclasts, bone forming osteoblasts, and mechanical sensing osteocytes. When individuals experience a disconnect in this tightly coupled process such that bone formation does not equal bone resorption, and/or both mechanisms are suppressed, fracture incidence increases (Hadjidakis & Androulakis, 2006). While therapeutic treatment options have significantly aided in the management of fracture, some patients still experience undesirable, adverse side-effects, and therefore, continued development of refined options is necessary (Bauer, 2018; Compston et al., 2019; Yedavally-Yellayi et al., 2019). As this quest continues, it is imperative to gain further insight in to the cellular and molecular responses occurring within the skeletal niche. Targeting metabolic pathways in bone cells is an incredibly provocative tool that could be applied to combat various conditions which lead to increased fracture incidence (i.e., post-menopausal osteoporosis, type 2 diabetes mellitus, and age-related osteoporosis). In fact, first-generation anti-resorptive bisphosphonates (i.e., etidronate and clodronate) used to treat osteoporosis inhibit cellular energy (adenosine triphosphate or ATP) of the osteoclast (Khosla et al., 2017). These widely prescribed drugs represent an impeccable example of how metabolic pathways can be exploited to impact overall bone health and improve patient quality of life. As such, the current study sought to provide an in-depth characterization of intracellular metabolites throughout osteoblast differentiation.

We conducted untargeted liquid chromatography tandem mass spectrometry (LC–MS/MS) based metabolomics investigation of primary murine bone marrow stromal cells (BMSCs) throughout stages of osteoblast differentiation including the stromal phase, committed osteo-progenitors, and mature matrix secreting osteoblasts (0, 2, and 7 days).

## Methods

### Bone marrow isolation and osteoblast culture

Primary, murine total bone marrow was isolated from the long bones (tibiae, femora, and iliac crest) of 7–10 week old male and female C57BL/6 N mice and plated for 48 h in complete α-MEM (α-MEM, 10% FBS, 1% penicillin/ streptomycin). In accordance with the plastic adherence theory, cells from the stromal/ mesenchymal lineage (BMSCs) adhered to the plastic culture ware whereas the non-adherent hematopoietic cells were washed away (Phinney et al., 1999; Sun et al., 2003). The adherent BMSCs were then collected following trypinsinzation, counted and plated in appropriate tissue-culture treated plates. BMSCs were then cultured in complete α-MEM or osteogenic medium (complete α-MEM, 50 µg/mL ascorbic acid, and 5 mM β-glycerol phosphate) to induce osteoblast differentiation for specified time point (Maridas et al., 2018). To demonstrate osteogenic potential and differentiation, cells were stained for alkaline phosphatase (ALP) enzymatic activity (red) and hematoxylin following 0, 2, 7 days, or Von Kossa at day 10 (Chung et al., 1992; Orriss et al., 2014; Sun et al., 2003).

### RNA isolation and gene expression analyses

Following BMSC isolation and culture described above, total RNA was isolated using ReliaPrep RNA Miniprep Systems (Promega, Madison, WI), according to the manufactures protocol. Following cDNA synthesis, each qPCR reaction was performed in duplicate using SYBR green chemistry (BioRad, Cat#1708882) on the BioRad CFX384Real-time system. All qPCR results were evaluated by the comparative cycle number at threshold (C_Q_) method (User Manual #2, Applied Biosystems), using hypoxanthine phosphoribosyltransferase 1 (*Hprt1*) as the invariant control, FWD 5′-GCC TAA GAT GAG CGC AAG TTG; REV 5′-TAC TAG GCA GAT GGC CAC AGG. Target gene primer sequences are as follows: *Runx2* FWD 5′-CGA CAG TCC CAA CTT CCT GT, REV 5′-CGG TAA CCA CAG TCC CAT CT; *Sp7* FWD 5′-GAA GTT CAC CTG CCT GCT CTG T, REV 5′-CGT GGG TGC GCT GAT GT; *Alpl* FWD 5′-GGT ATG GGC GTC TCC ACA GT, REV 5′-GCC CGT GTT GTG GTG TAG CT; *Col1a1* FWD 5′-CGT CTG GTT TGG AGA GAG CAT, REV 5′-GGT CAG CTG GAT AGC GAC ATC; *Bglap2* FWD 5′-TGA GCT TAA CCC TGC TTG TGA CGA, REV 5′-AGG GCA GCA CAG GTC CTA AAT AGT.

### Seahorse XFe flux analysis of ATP production

To measure ATP flux in real time, BMSCs were plated in Seahorse XFe 96-well plates at 2.0 × 10^4^ cells/well and cultured under osteogenic conditions for either 0, 2, or 7 days. On specified days, ATP assay were performed according to the manufacturers protocol. Briefly, basal assay medium was supplemented with 10 mM glucose, 1 mM sodium pyruvate, 2 mM glutamine, and 100 nM insulin. Subsequently, oligomycin (2 µM) and rotenone/antimycin A (1 µM/1 µM) were injected during assays while oxygen consumption rates (OCR) and extracellular acidification rates (ECAR) were monitored. Hoechst stain was also injected in the last port and a Cytation 5 (BioTek) was used provide cell counts, both for normalization and as a means to monitor proliferation throughout differentiation. Considering the stoichiometry of the glycolytic pathway, the rate of ATP produced via in glycolysis is calculated as such: glycolytic ATP production rate (pmol ATP/min) = glycolytic proton efflux rate (pmol H+/min). Conversely, the rate of oxygen consumption that is coupled to ATP production during oxidative phosphorylation can be calculated as the OCR that is inhibited by addition of the ATP synthase inhibitor, oligomycin: OCR_ATP_ (pmol O_2_/min) = OCR (pmol O_2_/min)—OCR_Oligo_ (pmol O_2_/min). Further transformation of OCR_ATP_ to the rate of mitochondrial ATP production consists of the final equation mitoATP production rate (pmol ATP/min) = OCR_ATP_ (pmol O_2_/min) × 2 (pmol O/pmol O_2_) × P/O (pmol ATP/pmol O). Finally, the total cellular ATP Production Rate is the sum of the glycolytic and mitochondrial ATP production rates: ATP Production Rate (pmol ATP/min) = glycoATP Production Rate (pmol ATP/min)+mitoATP Production Rate (pmol ATP/min).

### Sample preparation for metabolomics

Following indicated times of BMSCs in osteogenic medium, medium was removed from the cells, and washed twice with PBS. During the final was in PBS, cells were scraped and collected via centrifugation. Supernatant was removed from cell pellet and pellet was frozen at − 80 °C. The metabolite extraction method was performed as described previously (Trushina et al., 2013). Cells were thawed on ice at 4 °C followed by deproteinization with methanol (1∶4 ratio of extract to methanol) and vortexed for 10 s, followed by incubation at − 20 °C for 2 h. Prior to deproteinization, 4 µL of an internal standard solution of ^13^C_6_-Phenylalanine (247 ng/µL) was added to the cellular extracts, and plasma QC and pooled quality control (QC) samples to monitor the recovery of extracted metabolites. The samples were centrifuged at 18,000 g for 20 min at 4 °C. The supernatants were lyophilized (Savant, Holbrook, NY) and stored at − 20 °C prior to analysis or reconstituted in running solvents and analyzed within 24 h. Metabolite separation was performed using a 1200 Agilent UPLC system (Agilent Inc., USA) with both hydrophilic interaction chromatography (HILIC) (ethylene-bridged hybrid 2.1 × 150 mm, 1.7 mm; Waters) and reversed-phase liquid chromatography C18 (RPLC) (high-strength silica 2.1 × 150 mm, 1.8 µm; Waters) columns. For each column, the run time was 20 min at a flow rate of 400 µL/min. Reverse-phase chromatography was performed using 99% solvent A (5 mmol/L NH_4_ acetate, 0.1% formic acid, and 1% acetonitrile) to 100% solvent B (95% acetonitrile with 0.1% formic acid). The gradient was 0 min, 0% B; 1 min, 0% B; 3 min, 5% B; 13.0 min, 100% B; 16 min, 100% B; 16.5 min, 0% B; and 20 min, 0% B. The hydrophilic interaction chromatography gradient was as follows: 0 min, 100% B; 1 min, 100% B; 5 min, 90% B; 13.0 min, 0% B; 16 min, 0% B; 16.5 min, 100% B; and 20 min, 100% B. The injection volume was 5 µL and column was maintained at 50 °C. Pooled QCs and standards were run at the beginning and the end of each sequence to monitor shift in the retention time on the column.

### Mass spectrometry

Mass spectrometric acquisition was performed using a 6550 ToF–MS (Agilent Technologies) platform in both positive and negative electrospray ionization (ESI) modes, scan range of 50**–**1200 m/z. The mass accuracy and mass resolution were 5 parts per million (ppm) and 20,000 ppm, respectively. The instrument settings were as follows: nebulizer gas temperature 325 °C, capillary voltage 3.5 kV, capillary temperature 300 °C, fragmentor voltage 150 V, skimmer voltage 58 V, octapole voltage 250 V, cycle time 0.5 s, and run time 15 min. Final data represents n = 3 biological replicates and n = 3 technical replicates.

### Data preprocessing

All ToF–MS raw data files were converted to compound exchange File (.CEF) format using MassHunter DA Reprocessor software (Agilent Technologies Inc). Chromatography and centroided MS data were aligned to generate a single data matrix consisting of retention time (RT), mass-to-charge (m/z), and normalized ion intensity for each detected peak in individual samples. Mass profiler professional (Agilent Technologies Inc.) was used for data processing. Default settings were used with the exception of signal-to-noise ratio threshold, mass limit (0.0025 units), and time limit (9 s). The resulting metabolites were identified against the METLIN metabolite database using a detection window of ≤ 5 ppm. Putative identification of each metabolite was made based on mass accuracy (m/z), Kyoto encyclopedia of genes and genomes (KEGG) based identifiers. We annotated all metabolites reported in the study to level 2 as classified by the metabolomics standard initiative (MSI): putatively annotated compounds (without chemical reference standards) based on accurate precursor masses and mass spectral information by matching against spectral libraries (Sumner et al., 2007).

### Statistical processing of metabolomics and transcriptomics datasets

Statistical processing of both the combined metabolomics data sets was performed using statistical software R (Version 3.5.2) (Team, 2015). Normalized, transformed, imputed, outlier removed, and scaled peak area representative of relative metabolite amounts obtained from using DeviumWeb (Grapov, 2014) are presented. Hierarchical clustering analysis (HCA) was performed on Pearson distances using PermutMatrix (Caraux & Pinloche, 2004), where the data was normalized using z-scores of the relative abundance of the metabolites for heat map display. Correlations reported are Spearman rank correlations. Principal components analysis (PCA) and partial least squared discriminant analyses (PLSDA) were performed using MetaboAnalyst 4.0 (Chong et al., 2018) where the output displayed score plots to visualize the sample groups.

### Metabolic pathway and enrichment analysis of metabolomics datasets

For metabolomics datasets, pathway enrichment was performed using MetaboAnalyst 4.0 (www.metaboanalyst.ca) (Xia et al., 2015) and pathways presented are KEGG based.

### Data availability and sharing

All data discussed and presented in this manuscript are provided in Supplementary S1.

## Results

### Osteoblast differentiation and bioenergetics

**Fig. 1:**
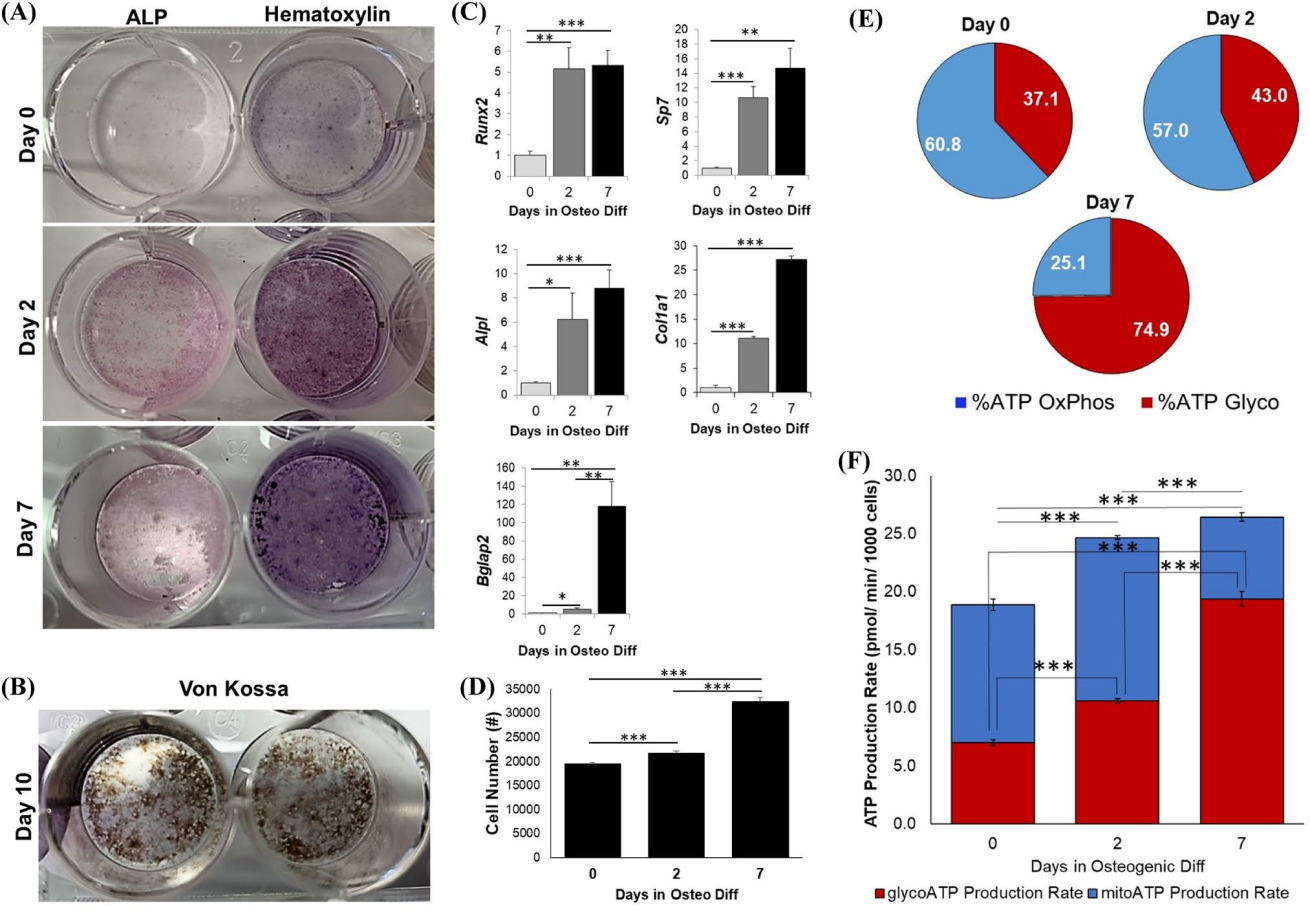
Characterization of osteoblast differentiation and bioenergetic profile. Primary, murine stromal cells throughout osteoblast differentiation stained for (**A**) alkaline phosphatase (ALP) enzymatic activity (red) or hematoxylin (blue/purple) at day 0, 2, and 7. **B** Von Kossa staining of mineralized matrix following 10 days of differentiation. **C** Gene expression analyses of yunt-related transcript factor (*Runx2*), osterix (*Sp7*), alkaline phosphatase (*Alpl*), type I collagen (*Col1a1*), and osteocalcin (*Bglap2*) normalized to *Hprt1*. **D** Cell counted following DAPI staining. **E** Metabolic pathway contribution for adenosine triphosphate (ATP) and **F** ATP production rates; blue, oxidative phosphorylation (OxPhos); red, glycolysis (Glyco). (*; *p* < 0.05, **; *p* < 0.01, ***; *p* < 0.001)

To demonstrate the osteogenic potential of murine BMSCs to osteoblasts, cells were stained with either ALP, an osteoblast-specific marker, or hematoxylin following 0, 2, or 7 days in osteogenic differentiation medium, or stained with Von Kossa to observe mineral deposition after 10 days. As expected, ALP is robustly expressed following 2 days in osteogenic medium and throughout osteoblast differentiation (Fig. 1A). Additionally, 10 days under osteogenic conditions these ‘mature’ osteoblasts have formed mineralized matrix as indicated by Von Kossa staining (Fig. 1B). Additional confirmation of osteogenesis is demonstrated by gene expression analyses whereby BMSC’s commit to the osteo-progenitor lineage by day 2 and more mature osteoblast markers are up-regulated by day 7 (Fig. 1C). Osteogenic differentiation also enhances proliferation as depicted by cell count numbers taken after 0, 2, or 7 days in culture (Fig. 1D). We next sought to characterize the metabolic profile of osteoblasts throughout differentiation. These data show that stromal cells (day 0) and osteo-progenitor cells (day 2) primarily generate ATP via oxidative phosphorylation, while mature osteoblasts (day 7) switch to a much more glycolytic phenotype (Fig. 1E, F). Interestingly, total ATP production also increases upon osteoblast differentiation (Fig. 1F).

### Metabolomics analysis for identification of individual metabolites and pathways

Using two different chromatographic separations (RPLC, and HILIC) in an untargeted manner using LC–MS/MS platform we obtained the relative abundances for a total 19,000–28,000 features (we defined them as a set of m/z, RT, MS1 intensity with a MS2 spectrum) obtained using LC–MS/MS analysis in both positive and negative modes. Combined (posHILIC, negHILIC, posRPLC, and negRPLC) together after consolidation of features, resulted in 1345 metabolites with relative abundances. The raw metabolite abundance values alongside the identified metabolite IDs, RTs, m/z values, formulae, InchiKeys, SMILES, and the raw relative abundance values are provided (Supplementary Table S1). Representative tandem MS/MS spectra for several compounds are also shown in Fig. 4. Further, these datasets were refined after normalization, transformation, and scaling, followed by imputation (Supplementary Table S2). Together these 1345 metabolites, were enriched for 60 KEGG-based metabolic pathways, of which pyrimidine metabolism, aminoacyl-tRNA biosynthesis, arginine biosynthesis, alanine, aspartate and glutamate metabolism, taurine and hypotaurine metabolism, histidine metabolism, beta-alanine metabolism, arginine and proline metabolism, linoleic acid metabolism, glutathione metabolism, and purine metabolism (all significantly enriched, P value < 0.05), in addition to hundreds of lipid classes that were also represented in these datasets but are not KEGG-pathway mappable.

**Fig. 2:**
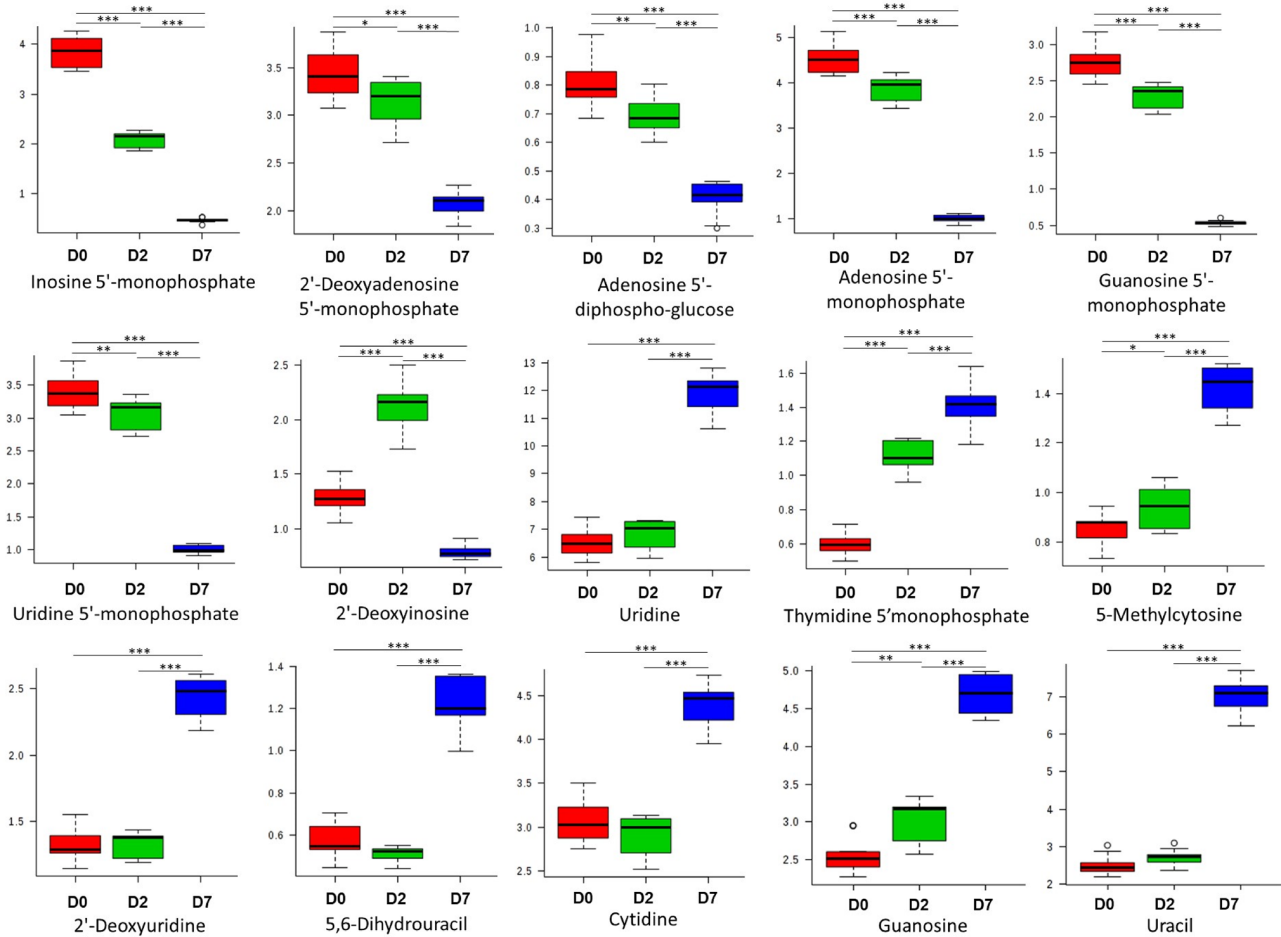
Individual nucleotide profiles in osteoblast differentiation. Significantly altered nucleotides throughout osteoblast differentiation at day 0, day 2, or day 7. (*; *p* < 0.05, **; *p* < 0.01, ***; *p* < 0.001)

**Fig. 3:**
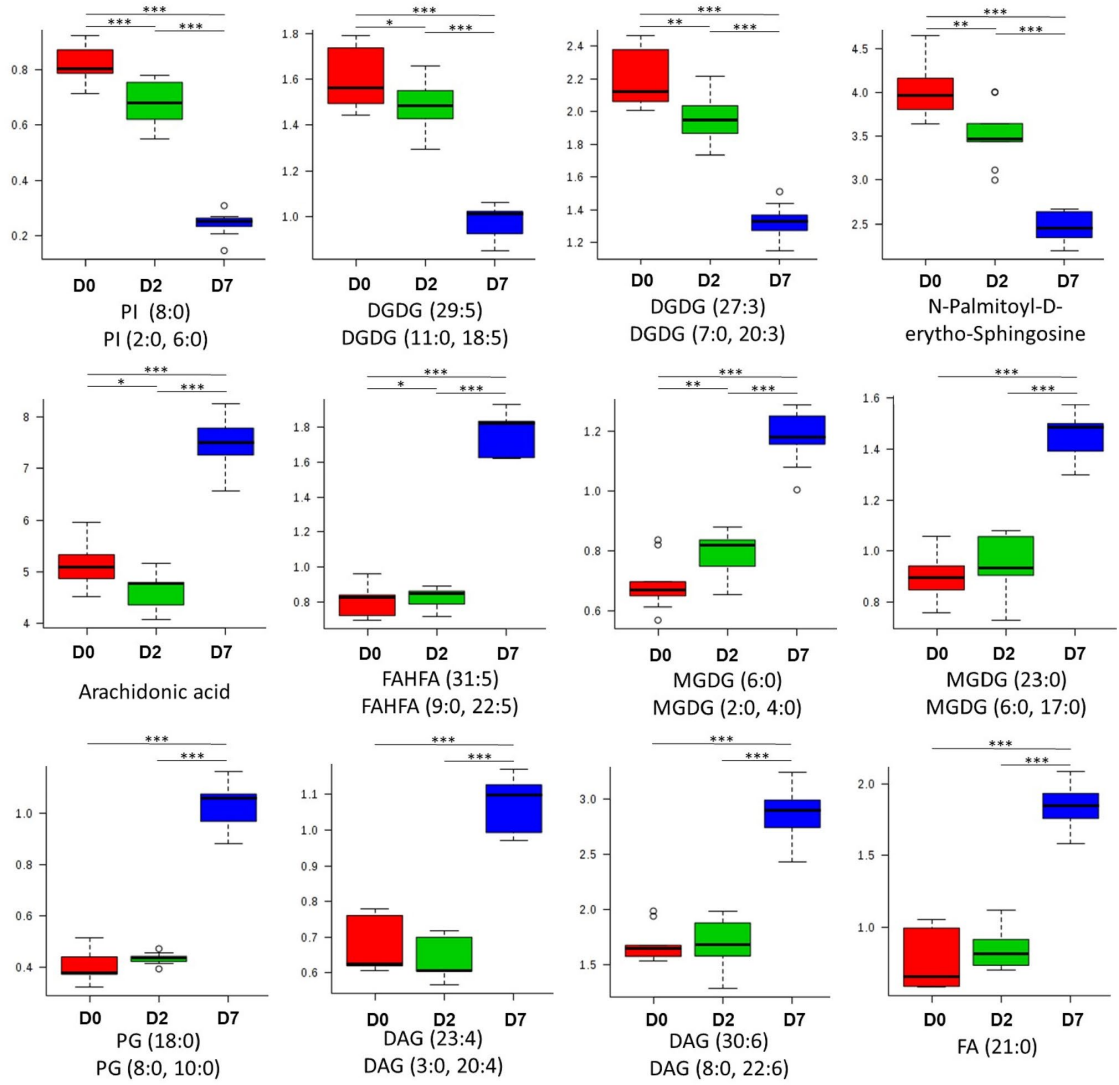
Lipid related metabolites altered throughout osteoblastogenesis. Significantly altered lipid-related metabolites throughout osteoblast differentiation at day 0, day 2, or day 7. *PI* phosphatidyl inositol, *DGDG* digalatosyldiacylglycerol, *MGDG* monogalatosyldiacylglycerol, *FAHFA* fatty acid esters of hydroxyl fatty acids, *PG* phosphatidylglycerol, *DAG* diacylglycerol, *FAs* fatty acids. (*; *p* < 0.05, **; *p* < 0.01, ***; *p* < 0.001)

**Fig. 4:**
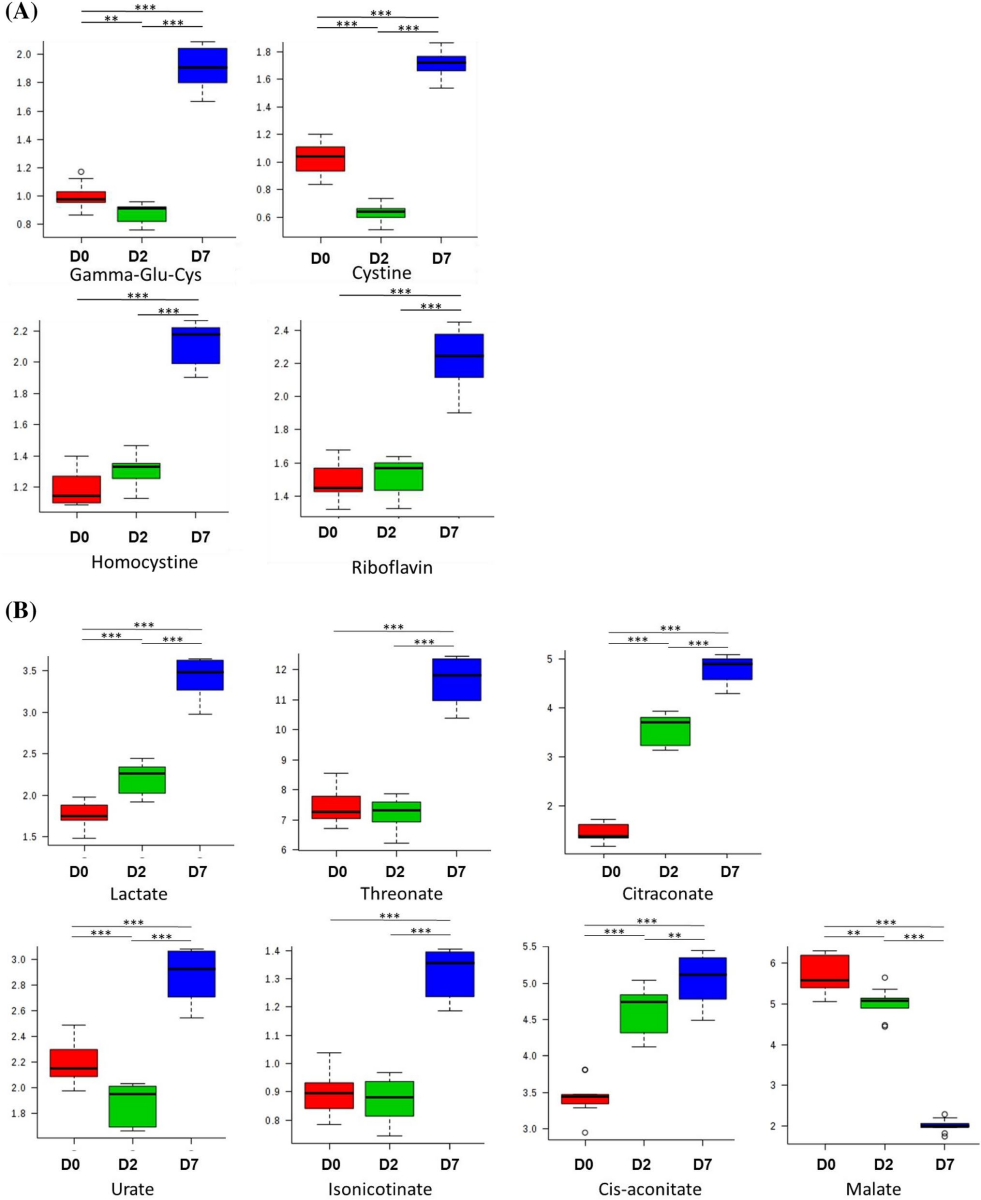
Metabolites involved in REDOX and TCA cycle. Significantly altered metabolites involved in (**A**) Redox metabolism, and (**B**) the TCA cycle throughout osteoblast differentiation at day 0, day 2, or day 7.ɤ-Glutamylcysteine (Gamma-Glu-Cys). (*; *p* < 0.05, **; *p* < 0.01, ***; *p* < 0.001)

Firstly, we looked at chemical classes showing interesting patterns during the time-course study based on their fold changes and statistically significant changes in abundances between a pair of conditions (Supplementary Table S3). We noted that specific purines, pyrimidines (i.e., I, A, G, U), and phosphates increased, while nucleotides U, T, C, G decreased with differentiation (Fig. 2). We also observed a decrease in several DGDGs, and an increase in MGDGs, and DAGs in the analysis (Fig. 3). Increasing trends from 0 d through 2 d to 7d was also observed for redox-associated metabolites such as gamma-glu-cys, cysteine, homocysteine, and riboflavin (Fig. 4A). Further, among the organic acids, except malate, we noted an increasing accumulation trend from 0 to 7 day (Fig. 4B).

### Multivariate analysis and markers derived thereof

**Fig. 5:**
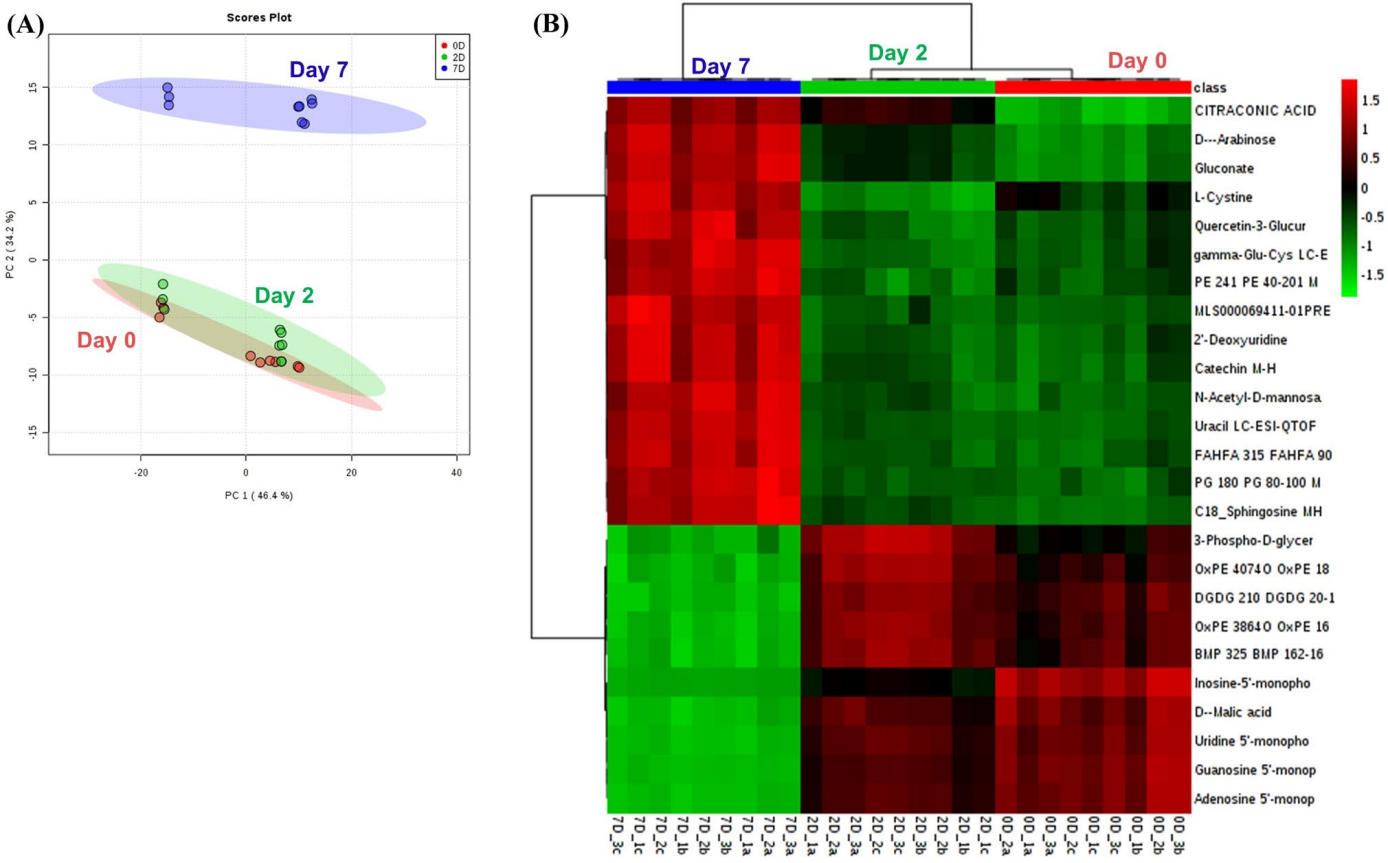
Distinct clustering of metabolites during osteoblasts differentiation. **A** PCA analysis and **B** HCA of intracellular metabolites from day 0, 2, and 7 of osteoblast differentiation

Figure 5A shows PCA score plot using an unsupervised multivariate analysis, principal component analysis (PCA) we observed that metabolite abundances alone were able to discriminate the 3 groups (0 d, 2 d and 7 d) which explained the variation in the dataset by virtue of the first 2 PCs (PC1, PC2) by 80.6%. Various metabolites that were identified as being responsible for the separation between the sample groups in a loading plot (data not shown), revealed the differentiating metabolites which included uridine, uracil, niacinamide, lysophosphatidylcholine 16:0, creatinine, threonic acid, catechin, inosine among others. Figure 5B shows heatmap from hierarchical clustering analysis (HCA) using Z-score normalized metabolite abundances of the quantified metabolites we observed clear clustering for the 3 time points, 0 d, 2 d and 7 d, as shown for top 25 metabolites obtained from an analysis of variance (ANOVA) analysis, where the upper cluster formed of metabolites where sugars (arabinose), redox metabolites (cysteine, quercetin-3-glucuronide, gamma-glucys, catechin) and several lipids (PE 24:1, FAHFA 31:5, PG:18:0, sphingosine) showed increased abundance for 7 d samples, when compared to 0 d and 2 d cells. The bottom cluster formed with low metabolite abundances in 7 d cells when compared against 0 d and 2 d samples, and these metabolites are mostly nucleoside phosphates (IMP, UMP, GMP, AMP), lipids (OxPE 40:7, DGDG 21:0, OxPE 38:6, BMP 32:5) and malate.

**Fig. 6:**
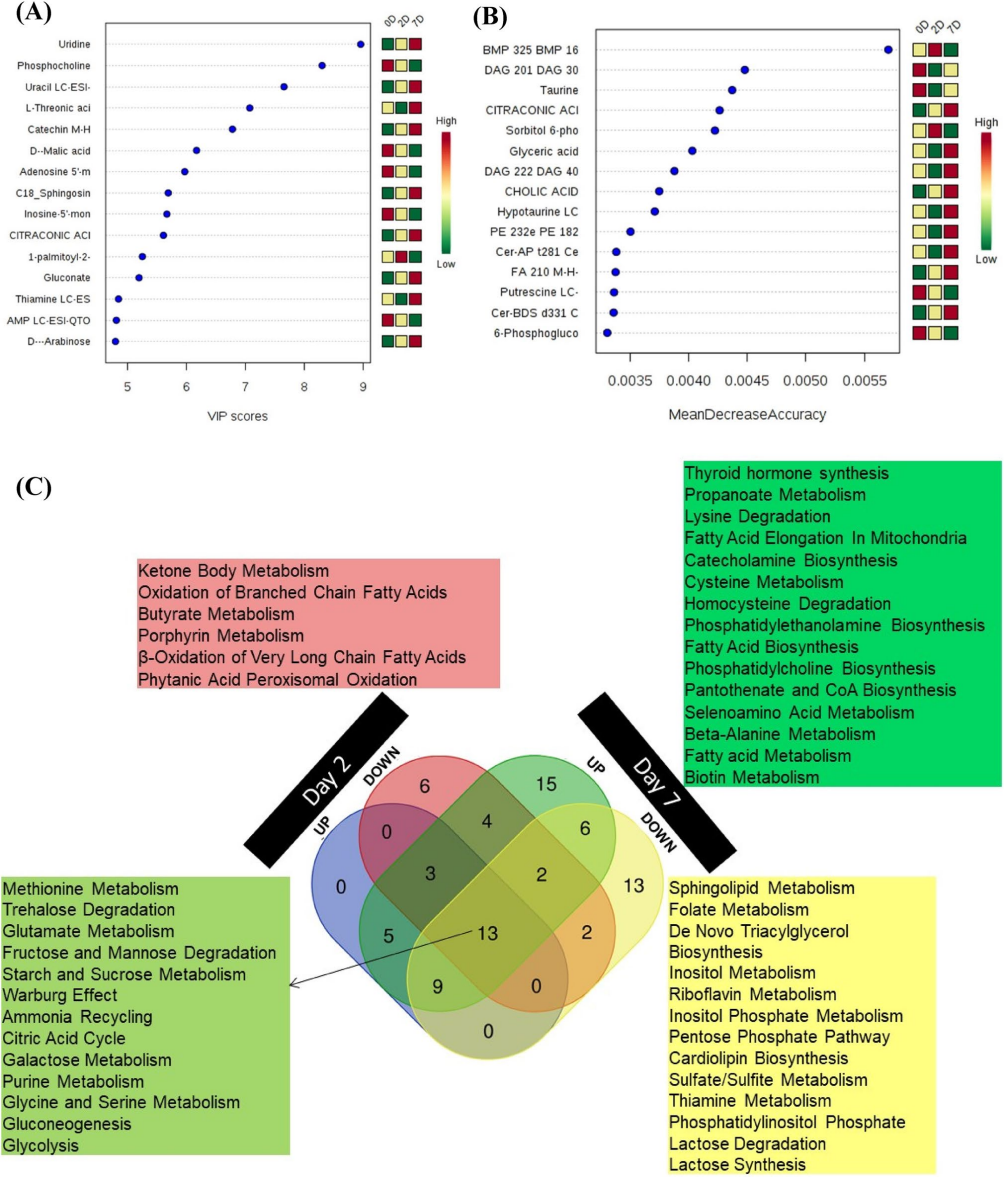
Osteogenic biomarkers (**A**) Variable importance in projection (VIP) contribution of the differentiation biomarker metabolites for the differential expression among groups. **B** Top 15 metabolites with the strongest influence on the prediction accuracy (i.e., mean decrease accuracy, MDA) of the random forest (RF) analysis are presented in order of importance (top to bottom). RF analysis used individual metabolite profiles to predict whether the samples were from 0, 2, or 7 days of osteoblast differentiation. **C** Vin diagram of significantly (P value < 0.05) increased (FC > 1.2) and decreased (FC < 0.8) metabolites in 2-day and 7-day cells when compared to 0-day cells. Colors in the vin diagram correspond to various metabolic pathways listed in similarly colored pathway list

In order to identify the genes responsible for the discrimination among the metabolomic profiles, the Variable importance in projection (VIP) score derived from the partial least square discriminant analysis (PLS-DA) model was used to select those with the most significant contribution in a PLS-DA model. VIP scores are a weighted sum of PLS weights for each variable and measure the contribution of each predictor variable to the model. Further, the VIP statistic summarizes the importance of the metabolites in differentiating the study groups (i.e., 0 d, 2 d, and 7 d) in multivariate space (Smart & Hodgson, 2018). The compounds exhibiting the higher VIP score are the more influent variables. Our VIP analysis revealed, purines/pyrimidines (uridine, uracil, adenosine-5′-monophosphate, inosine-5′-monophosphate), phosphocholine, organic acids (threonate, malate, citraconate, gluconate), arabinose, sphingosine, thiamine and catechin among others were the major contributors (Fig. 6A).

Random forest (RF) analysis is a data-driven method designed for prediction and is conducted to identify metabolites that improved prediction of time points or the 3 sample groups. Contributions of individual predictors are measured by ‘variable importance’ using a conditional permutation scheme for correlated predictors. Variable importance greater (less) than 0 suggests an increase (decrease) in prediction accuracy. By relying on the ranges of values of each selected feature using our RF classifier, one can identify dependencies between features which results in a good separation for the two classes of interest, to help identify the most important metabolites and to exclude associations by chance. Thus, from RF analysis we present the top 15 features based on mean importance of each feature that were several lipids (BMP 32:5, DAG 20:1, DAG 22:2, PE 23: 2, Cer-AP 28:1, FA 21:0, Cer-BDS 33:1), organic acids (cholate, citraconate, glycerate), redox metabolites (taurine, hypotaurine), putrescine among others (Fig. 6B). Finally, we looked at the significantly (P value < 0.05) increased (FC > 1.2) and decreased (FC < 0.8) metabolites in day 2 and day 7 cells when compared to day 0 osteo-progenitor cells (Fig. 6C). This revealed that there were profoundly more metabolites increased and decreased in mature osteoblasts (7 days) than cells differentiated for 2 days, when both compared to day 0 time point (Fig. 6C).

## Conclusions

The data presented here highlights the intracellular metabolites present throughout osteoblast differentiation process. Importantly, these data are from primary murine stromal cells, which captures the early stages of osteoblast differentiation and represents physiological relevance. This is different to other studies within the field which detail phenotypes associated with committed osteoblasts as with commonly used cell lines and calvaria cells (Esen et al., 2013; Guntur et al., 2014; Lee et al., 2020). Therefore, these data are of relevance in the field of bone biology in targeting bone formation via osteoblast differentiation processes.

To be expected, our data demonstrate that committed, mature osteoblasts (day 7) produce ATP primarily through glycolysis (~ 75%) compared to oxidative phosphorylation (~ 25%), which has been previously described as aerobic glycolysis or the Warburg effect (Esen et al., 2015; Guntur et al., 2014). Consistent with this glycolytic profile, mature osteoblasts increase lactate production compared to cells at early stages of differentiation. Interestingly, stromal cells and early, osteo- progenitors demonstrated a striking oxidative profile whereby ATP was primarily generated via oxidative phosphorylation (~ 60%), which has only recently been described but in calvaria osteoblasts (Lee et al., 2020). Upon osteoblast differentiation, nucleotides were differentially impacted, however, its speculated that RNA synthesis and transcription is upregulated as the methylated form of DNA, 5′-methylcytosine, is enhanced in mature osteoblasts, consistent with other studies in various cell models (Kang et al., 2007; Locklin et al., 1998; Zhang et al., 2011). Additionally, lipid metabolism, which until recently has been generally underappreciated in the field (Rendina-Ruedy et al., 2017), is also distinctly regulated during the osteoblast differentiation process. Early in the differentiation process, stromal and osteo-progenitor cells demonstrate high levels of lipid metabolites reflective of membrane synthesis, which drop in more mature cells. This is speculated to be reflective of the initial proliferative phase stromal cells experience upon osteogenic differentiation. Conversely, lipid signaling molecules, such as arachidonic acid, FAHFA, DAGs, are highly enriched in mature osteoblasts. Additionally, we noted a distinct enrichment of metabolites associated with oxidative stress including gamma-glu-cys, cysteine, homocysteine, and riboflavin in mature osteoblasts. This is of particular interest given that these cells have reduced oxidative phosphorylation, which would indicate potentially less reactive oxygen species (ROS) generated from the mitochondria. However, others have noted that osteoblast differentiation is associated with enhanced ROS production (Arakaki et al., 2013), while our group has documented lipid droplets at this stage in osteoblast differentiation (Rendina-Ruedy et al., 2017). Taken together, these data would indicate that osteoblasts are particularly sensitive to ROS and could be using lipid droplets as a mechanism to maintain redox homeostasis and protect against lipotoxicity by sequestering toxic lipids (Herms et al., 2013). Finally, these metabolic data demonstrate an increase in some tricarboxylic acid cycle (TCA) cycle intermediates in mature osteoblasts, most notably cis-isocitrate, the intermediate of citrate to isocitrate. In addition to being a ‘TCA cycle intermediate’, citrate is both an incredibly important component of the mineralized portion of bone, the hydroxyapatite, secreted by osteoblasts (Hu et al., 2010), and can also be shuttled out of the mitochondria for fatty acid/ sterol synthesis. While threonate is also elevated in mature osteoblasts, threonate is the active metabolite of ascorbic acid, which is a critical component of the osteogenic medium, our differentiation cocktail is responsible. A final note related to TCA cycle, is that malate is remarkably high in early stromal cells and osteoprogenitors compared to mature osteoblasts. As malate is one of the final metabolites used in the TCA cycle to produce oxaloacetate, this is consistent with high oxidative phosphorylation noted in these cells, but also demonstrates a disconnect in the cycle in mature osteoblasts.

In summary, the current study used primary, murine BMSCs differentiated to osteoblasts, and provides a rigorous profile of intracellular metabolites through this process. These data are expected to provide novel insight as to how alterations in metabolic processes regulate osteoblastogenesis, and subsequent bone formation.

## Supplementary Information

Below is the link to the electronic supplementary material.

Supplementary file1 (XLSX 436 kb)

Supplementary file2 (XLSX 370 kb)

Supplementary file3 (XLSX 486 kb)

Supplementary file4 (XLSX 1445 kb)

## Declarations

### Research involving human and animal participants

All experimental procedures were approved by the Institutional Animal Care and Use Committee (IACUC) at the Maine Medical Center Research Institute and Vanderbilt University Medical Center. All applicable international, national, and/or institutional guidelines for the care and use of animals were followed.
